# Dataset on the physicochemical properties of raw and torrefied tropical hardwood sawdust under varying torrefaction conditions

**DOI:** 10.1016/j.dib.2025.112438

**Published:** 2026-01-07

**Authors:** Noor Asma Fazli Abdul Samad, Suriyati Binti Saleh

**Affiliations:** Faculty of Chemical and Process Engineering Technology, Universiti Malaysia Pahang Al-Sultan Abdullah, Lebuh Persiaran Tun Khalil Yaakob, 26300 Kuantan Pahang Malaysia

**Keywords:** Mass yield, Energy yield, High heating value, Ultimate analysis, Proximate analysis, H/C ratio, O/C ratio

## Abstract

This article presents a dataset on the physicochemical properties of tropical hardwood sawdust subjected to torrefaction under different process conditions. The dataset includes measurements for raw sawdust and samples torrefied at varying temperatures and durations. Key parameters reported are mass yield, energy yield, higher heating value (HHV), enhancement factor of HHV, proximate analysis (volatile matter, fixed carbon, and ash content), ultimate analysis (C, H, N, O, and S content), and elemental ratios (H/C and O/C). The data were collected using standard analytical methods for biomass characterization. Torrefaction was conducted at controlled temperatures ranging from 240 °C to 330 °C for residence times of 15–60 min. Mass and energy yields were calculated based on the initial and final sample weights and calorific values. The HHV was determined using a bomb calorimeter, while proximate analysis was carried out according to ASTM procedures. Ultimate analysis was performed using a CHNS analyzer, and oxygen was estimated by difference. Atomic ratios were calculated to provide insights into fuel quality changes induced by torrefaction. The dataset is presented in tabular format, allowing easy integration into further statistical, thermodynamic, or modeling studies. It provides a valuable reference for researchers working on biomass upgrading, energy conversion, and sustainable fuel development. In particular, the data can support the development of empirical and predictive models for biomass torrefaction, facilitate comparative analysis with other feedstocks, and contribute to life cycle or techno-economic assessments of biomass-to-energy pathways. The dataset is hosted in an open-access format, ensuring reusability for energy engineers, material scientists, and policy researchers interested in renewable energy and biomass valorization.

Specifications Table

**Table:** 

Subject	Engineering & Materials science
Specific subject area	Biomass torrefaction, fuel characterization and thermochemical conversion
Type of data	Table
Data collection	Tropical hardwood sawdust (Seraya, Kulim, Meranti, and Chengal) collected from a sawmill in Gambang, Pahang, Malaysia was torrefied in a tubular reactor at 240–330 °C for 15–60 min. Physicochemical properties of raw and torrefied samples were measured: proximate analysis (volatile matter, ash, fixed carbon) followed ASTM E872–82 and ASTM 1755–01, HHV was determined using a bomb calorimeter (C200, IKA® Works, Asia Sdn Bhd), and ultimate analysis (C, H, N, S, O) was conducted with a CHNS/O analyzer (FlashSmart, Thermo Scientific). All experiments were carried out in triplicate and average values reported.
Data source location	Faculty of Chemical and Process Engineering Technology, Universiti Malaysia Pahang Al-Sultan Abdullah, Lebuh Persiaran Tun Khalil Yaakob, 26300 Kuantan Pahang Malaysia
Data accessibility	Repository name: Mendeley Data Data identification number: doi:10.17632/v7gcxxwmst.1 Direct URL to data: https://data.mendeley.com/datasets/v7gcxxwmst/1
Related research article	None

## 1 Value of the Data

• This dataset provides comprehensive measurements of mass yield, energy yield, higher heating value (HHV), proximate and ultimate composition, and atomic ratios for raw and torrefied tropical hardwood sawdust (Seraya, Kulim, Meranti, and Chengal). Such data for tropical hardwood species remain limited compared to temperate softwoods, enabling meaningful feedstock-specific comparisons under varying torrefaction conditions.
• The dataset enables evaluation of how torrefaction temperature and residence time influence fuel quality, energy density, and combustion-related properties of tropical hardwood sawdust. These data support the development of correlations and standardized approaches for upgrading biomass into more uniform solid biofuels, with direct relevance to co-firing and bio-pellet applications.
• By providing multiple physicochemical parameters across a defined range of torrefaction severities, the dataset is well suited for calibrating and validating predictive models of biomass torrefaction and combustion. It can support empirical, kinetic, thermogravimetric, and data-driven modeling studies involving tropical biomass materials.
• The dataset contributes to benchmarking tropical hardwood sawdust against other biomass feedstocks for thermochemical conversion pathways such as co-firing, gasification, and pyrolysis, thereby broadening the applicability of biomass-to-energy research across different regions.
• The dataset can be integrated into life cycle assessment (LCA) and techno-economic analyses by providing reliable experimental values for energy content and yields under controlled torrefaction conditions, supporting sustainability and biomass valorization studies.
• All experiments and measurements were conducted in triplicate and reported as mean values using standardized analytical methods, ensuring data reliability and reproducibility and supporting reuse across experimental, modeling, and industrial applications.

## 2 Background

The motivation for compiling this dataset arises from the large quantities of wood sawdust generated as by-products of sawmilling activities. According to national assessments, Malaysia generates approximately 3.6–4.2 million tonnes of woody biomass annually, of which about 1.94 million tonnes originate from wood-based processing industries in the form of sawdust, offcuts, slabs and shavings [1]. This highlights the substantial availability of sawdust residues and underscores their relevance as a representative biomass resource in the Malaysian context. Tropical hardwoods such as Seraya, Kulim, Meranti and Chengal are widely used in Malaysia for furniture, interior joinery, paneling, mouldings, plywood, and boatbuilding. The milling of these species produces substantial sawdust residues which are often disposed of through landfilling or open burning. Such practices not only contribute to environmental pollution but also result in the loss of valuable biomass resources. Although wood sawdust has potential as a renewable energy source but its direct use as fuel is restricted by inherent drawbacks including high moisture content, low bulk density, and relatively low higher heating value (HHV). These limitations reduce combustion efficiency and complicate handling, storage and transport processes which highlight the need for pretreatment strategies to improve its fuel quality. To address this challenge, sawdust from Seraya, Kulim, Meranti and Chengal undergoes torrefaction as a pretreatment under controlled conditions across a range of temperatures and residence times. The dataset was compiled to systematically record the resulting physicochemical changes including proximate and ultimate composition, HHV, mass and energy yields as well as elemental ratios. Standardized methodologies and calibrated instruments were employed with all experiments and measurements performed in triplicate to ensure reproducibility. This dataset provides a structured and open-access reference for researchers and practitioners working on biomass upgrading pathways and supports the broader transition toward sustainable energy generation from forestry residues.

## 3 Data Description

This dataset in the file *Data Physicochemical Raw and Torrefied Wood Sawdust.xlsx* contains information on the physicochemical properties of wood sawdust samples derived from four tropical hardwood species namely Seraya, Kulim, Meranti, and Chengal. The samples underwent torrefaction as pretreatment under controlled conditions. Operating conditions included temperatures of 240, 270, 300 and 330 °C and residence times of 15, 30, and 60 min. Raw samples were also included as baselines. The dataset reports the following parameters which are mass yield ( %), energy yield ( %), higher heating value (HHV, MJ/kg), enhancement factor of HHV, proximate analysis (volatile matter, fixed carbon, ash content on a dry basis), ultimate analysis (C, H, N, O, S in wt %), and derived H/C and O/C atomic ratios. All experiments were performed in triplicate and all values reported in the dataset represent the mean of three independent measurements. Standard deviations are not included since the dataset is intended to provide representative average physicochemical properties under defined torrefaction conditions. In total, the dataset comprises 52 entries (4 wood species × 12 torrefaction conditions + 4 raw references). Each entry corresponds to a unique combination of species and treatment condition enabling systematic evaluation of physicochemical changes due to torrefaction.

## 4 Experimental Design, Materials and Methods

### 4.1 Tropical hardwood samples preparation

In this study, tropical hardwood sawdust from Seraya, Kulim, Meranti, and Chengal was collected from a sawmill in Gambang, Pahang, Malaysia. The collected wood sawdust samples were first air-dried under ambient laboratory conditions (25–30 °C) for 3 days to reduce moisture content. Subsequently, the samples were oven-dried at 105 °C for 10 h using a laboratory drying oven (Model OMS60, Thermo Scientific Oven) until the moisture content was below 15 % thereby preventing microbial degradation and rotting. After drying, the material was ground using a grinding machine and sieved, with particles in the range of 0.5–1 mm collected to ensure the effectiveness of the torrefaction process [2].

### 4.2 Torrefaction experiment

The torrefaction process was conducted using a stainless-steel vertical tubular reactor with an internal diameter of 1.9 cm and a length of 39.7 cm as described in the previous study [3]. A layer of glass wool was placed at the base of the reactor to support the biomass and prevent sample loss. Approximately 10 g of sawdust was prepared and loaded into the reactor for each run. To maintain anoxic conditions and prevent ignition or oxidation, the reactor was purged with nitrogen gas at a flow rate of 10 mL/min for 5 min before heating. Torrefaction was carried out at temperatures of 240, 270, 300, and 330 °C with residence times of 15, 30, and 60 min. The reactor was heated at a constant heating rate of 10 °C/min using an electric furnace to target temperatures of 240, 270, 300, and 330 °C, after which each target temperature was maintained for the specified residence time. The internal reactor temperature was monitored using a K-type thermocouple positioned 19.5 cm from the top of the reactor. Afterwards, the reactor was allowed to cool naturally to room temperature, and the torrefied samples were collected, weighed, and stored in airtight containers for subsequent physicochemical characterization.

### 4.3 Analysis of raw and torrefied tropical hardwood sawdust

Mass and energy yields are important parameters for evaluating the torrefaction process. Mass yield represents the percentage of biomass retained after torrefaction, while energy yield indicates the proportion of total energy preserved following the process. Both parameters were calculated using (1), (2) [4].

(1) $$Mass\;Yield\;\left(\%\right)=\frac{Mass\;of\;Torrefied\;Sample\;\left(g\right)}{Mass\;of\;Raw\;Sample\;\left(g\right)}\times \;100\%$$

(2) $$Energy\;Yield\;\left(\%\right)=Mass\;Yield\times \frac{HHV\;of\;Torrefied\;Sample}{HHV\;of\;Raw\;Sample}$$

The higher heating value (HHV) is defined as the energy released during complete combustion and represents the maximum recoverable energy from biomass sources. The HHV of raw and torrefied samples was determined using an oxygen bomb calorimeter (C200, IKA® Works, Asia Sdn Bhd). For each measurement, approximately 0.5–1 g of sample was used. The enhancement factor of HHV is important indicator for evaluating torrefaction performance and was calculated using Eq. (3) [5].

(3) $$Enhancement\;Factor\;of\;HHV=\frac{HHV\;of\;Torrefied\;Sample}{HHV\;of\;Raw\;Sample}$$

Proximate analysis (volatile matter, ash and fixed carbon) was performed according to ASTM E872–82 and ASTM 1755–01 [2]. Meanwhile, ultimate analysis (C, H, N, S and O (by difference)) was carried out using a CHNS/O analyzer (FlashSmart, Thermo Scientific). All measurements were conducted in triplicate and the reported values represent averages. The hydrogen-to-carbon (H/C) and oxygen-to-carbon (O/C) ratios were subsequently calculated using (4), (5).

(4) $$H/C\;Ratio=\frac{\left(\frac{Mass\;Fraction\;of\;Hydrogen}{Atomic\;Weight\;of\;Hydrogen}\right)}{\left(\frac{Mass\;Fraction\;of\;Carbon}{Atomic\;Weight\;of\;Carbon}\right)}$$

(5) $$O/C\;Ratio=\frac{\left(\frac{Mass\;Fraction\;of\;Oxygen}{Atomic\;Weight\;of\;Oxygen}\right)}{\left(\frac{Mass\;Fraction\;of\;Carbon}{Atomic\;Weight\;of\;Carbon}\right)}$$

All experiments and measurements were performed in triplicate under identical conditions, and the reported values correspond to the mean of the three measurements without standard deviation.

## Limitations

Several limitations are associated with the dataset. Firstly, the study focused on four selected tropical hardwood species (Seraya, Kulim, Meranti, and Chengal) which may limit the generalizability of the data to other types of biomass or forestry residues. Secondly, the experiments were conducted under a defined range of torrefaction conditions (240–330 °C and 15–60 min). While these conditions are representative of typical torrefaction operations but data outside these ranges are not included. Thirdly, the dataset represents average values obtained from triplicate measurements but variations at larger scales or in industrial settings may differ due to heterogeneity of biomass feedstocks. Fourth, the oxygen content in ultimate analysis was estimated by difference which may introduce minor uncertainty. Finally, the data size is limited to 52 entries which provides a comprehensive overview for the selected species but may not fully capture all variability in tropical hardwood residues. Despite these limitations, the dataset remains a reliable and valuable reference for researchers and practitioners which offering structured and reproducible data for biomass torrefaction studies and renewable energy applications

## Ethics Statement

All authors have read and follow the ethical requirements for publication in Data in Brief and confirming that the current work does not involve human subjects, animal experiments, or any data collected from social media platforms.

## Credit Author Statement

**Noor Asma Fazli Abdul Samad**: Conceptualization, Methodology, Visualization, Investigation, Validation, Writing- Reviewing and Editing. **Suriyati Binti Saleh**: Biomass collection and preparation, Experimental works, Data curation, Writing, Original draft preparation.

## Declaration of generative AI and AI-assisted technologies

During the preparation of this work, the authors used ChatGPT in order to polish language and grammar as well as rephrase sentences to improve clarity. After using this tool, the authors reviewed and edited the content as needed and takes full responsibility for the content of the published article.

## Data Availability

Mendeley DataData Physicochemical Raw and Torrefied Wood Sawdust (Original data)
